# Challenges and prospects in mentoring medical scientists in Latin America: a narrative review and recommendations

**DOI:** 10.3389/fmed.2025.1595325

**Published:** 2025-07-16

**Authors:** Rodrigo C. Menezes, Caian L. Vinhaes, Felipe Ridolfi, Isabella B. B. Ferreira, Moises A. Huaman, Cesar Ugarte-Gil, Bruno B. Andrade

**Affiliations:** ^1^Laboratório de Pesquisa Clínica e Translacional, Instituto Gonçalo Moniz, Fundação Oswaldo Cruz, Salvador, Brazil; ^2^Multinational Organization Network Sponsoring Translational and Epidemiological Research (MONSTER) Initiative, Salvador, Brazil; ^3^Instituto de Pesquisa Clínica e Translacional, Faculdade Zarns, Clariens Educação, Salvador, Brazil; ^4^Departamento de Infectologia, Hospital das Clínicas, Faculdade de Medicina da Universidade de São Paulo, São Paulo, Brazil; ^5^Division of Infectious Diseases, Department of Medicine, Vanderbilt University Medical Center, Nashville, TN, United States; ^6^Instituto Nacional de Infectologia Evandro Chagas, Fundação Oswaldo Cruz, Rio de Janeiro, Rio de Janeiro, Brazil; ^7^Programa de Pós-Graduação em Medicina e Saúde Humana, Escola Bahiana de Medicina e Saúde Pública, Salvador, Brazil; ^8^Department of Internal Medicine and Center for Clinical and Translational Science and Training, University of Cincinnati College of Medicine, Cincinnati, OH, United States; ^9^Department of Epidemiology, School of Public and Population Health, University of Texas Medical Branch, Galveston, TX, United States

**Keywords:** medical education, research training, Latin America, leadership, funding

## Abstract

**Objective:**

To identify the challenges for the development and mentorship of physician-scientists in Latin America, outline the key barriers to integrating research training into medical education and post-graduate pathways, and propose recommendations to foster structured mentorship, improve institutional support, and strengthen the research ecosystem for future physician-scientists.

**Study design:**

Narrative review.

**Methods:**

A narrative review of relevant literature, institutional reports, and existing educational models was undertaken. The authors synthesized information on current educational pathways, funding opportunities, institutional infrastructures, and mentorship practices for physician-scientists in Latin America and derived practical recommendations for improving mentorship and training structures.

**Results:**

Latin America’s aspiring physician-scientists face fragmented educational programs, limited or inconsistent research integration in medical curricula, and inadequate mentorship support. These issues are exacerbated by overburdened healthcare systems, insufficient funding, low stipends, and a scarcity of dual-degree training programs. Institutional and funding barriers frequently force trainees and established researchers to choose between clinical practice and research, stifling the growth of a skilled physician-scientist workforce. Proposed solutions include developing integrated dual-degree and research residency programs, reforming medical curricula to include foundational research skills, strengthening mentorship networks through structured support and incentives, increasing research fellowship funding and removing exclusivity clauses, and creating regional consortia to standardize training and foster cross-border collaboration.

**Conclusion:**

Addressing the systemic barriers to mentoring and training physician-scientists in Latin America is essential for building a robust, research-oriented medical community. The recommended structural reforms, with increasing funding, enhanced mentorship frameworks, and promoting regional partnerships, can help Latin America cultivate a sustainable environment for the development of physician-scientists.

## 1 Introduction

Physician-scientists possess a unique skill set that combines clinical expertise with the ability to conduct research, allowing them to translate scientific discoveries into practical solutions for health challenges. These professionals, medical doctors with or without additional degrees who dedicate significant effort into research activities, play a vital role in addressing the diverse health needs of the Latin American population (1, 2). The region faces a broad spectrum of infectious diseases, like dengue and tuberculosis, and non-communicable diseases, such as diabetes and cardiovascular conditions, challenges that are compounded by profound socioeconomic, political, and environmental barriers that hinder healthcare access and equity. Developing sustainable, locally driven solutions to these complex issues demands a robust and well-supported physician-scientist workforce.

However, the training ecosystem for physician-scientists in Latin America remains fragmented and under-resourced. Although undergraduate research programs and postgraduate fellowships are available in many countries, these opportunities are often concentrated in capital cities and better-resourced universities, leaving large segments of the population underserved. Mentorship, an essential element in cultivating research skills and scientific identity, remains informal, unevenly distributed, and under-supported. These limitations are compounded by low stipends, lack of protected research time, and weak institutional incentives, which collectively discourage sustained engagement in research careers.

Globally, countries like the United States, Germany, the United Kingdom, and France demonstrate how structured educational pathways, dedicated funding, and institutional support can foster physician-scientists (1–5). In contrast, Latin America’s path is marked by a mismatch between the available infrastructure and the scale of demand for research training and career development within the region (6, 7). A few well-established institutions have made important contributions, but these remain isolated examples within a broader landscape of systemic underinvestment and policy gaps (8–10).

This narrative review examines the current landscape for mentoring physician-scientists in Latin America, identifying key obstacles across the training continuum and offering recommendations to build more inclusive, coordinated, and sustainable career pathways.

## 2 Methods

This is a narrative review based on a purposive selection of relevant peer-reviewed literature, institutional reports, regional policy documents, and educational program descriptions. Literature was identified through searches in PubMed, SciELO, and Google Scholar using combinations of keywords such as physician-scientist, mentorship, medical education, Latin America, and research training. Additional gray literature and institutional publications were included to reflect recent developments in national programs and funding strategies. The focus was primarily on materials published in the past 10 years, though foundational or widely cited documents outside this range were also considered when relevant. Sources were selected to capture both academic and policy perspectives on physician-scientist training in the region.

## 3 Educational pathways and challenges

### 3.1 The undergraduate path to physician-scientist

The journey to becoming a physician-scientist in Latin America often starts with a six- to 7-years undergraduate medical program. While countries like Argentina, Mexico, and Chile share similar structures, the integration of research into medical training remains inconsistent across the region. Consequently, aspiring physician-scientists often have limited exposure to research, as many medical programs lack formal research training within their curricula, leaving students without the necessary foundational skills and structured guidance.

In Brazil, for example, scientific initiation programs funded by the National Council for Scientific and Technological Development (CNPq) have long served as the main entry point, introducing thousands undergraduates to research (11). However, the absence of a harmonized curriculum, limited institutional incentives, and insufficient infrastructure weaken the impact of these efforts. Effective mentorship is a fundamental but fragile component in these programs, heavily reliant on the commitment of mentors who receive minimal institutional support and resources. The lack of a structured educational framework leads to inconsistencies in research training quality, which largely depends on each mentor’s availability, experience, and dedication. Moreover, the absence of formal mechanisms for collecting and acting on feedback from mentees prevents the identification of gaps in supervision, hinders the continuous improvement of training practices, and perpetuates unequal learning experiences across institutions.

Similar initiatives aimed at exposing undergraduates to research exist throughout Latin America. Peru offers Prociencia, Uruguay has the National Agency for Research and Innovation, and Argentina operates through its National Scientific and Technical Research Council. However, these programs vary widely in their effectiveness due to differences in funding, institutional support, and the availability of qualified mentors. Students in smaller cities or at under-resourced universities face additional obstacles in finding engaged mentors and accessing meaningful research experiences, which further limits their development as future physician-scientists.

For highly motivated students, that thrive despite challenges, the pursuit of research often means independently seeking opportunities, such as participating in extracurricular projects or securing international opportunities. Yet, even when students succeed in finding opportunities, formal dual-degree programs like MD-PhD pathways remain rare across Latin America. Consequently, many students see their research training interrupted upon graduation as they confront new obstacles and must choose between a clinical or research career.

This gap is particularly evident when compared to the United States, where MD-PhD programs offer a dual pathway for clinical and research training from the outset (12). For instance, the National Institutes of Health in the U.S. supports numerous MD-PhD programs, with thousands of physician-scientists affiliated with the program (13). In Colombia, the Universidad de Los Andes launched an MD-PhD program but it remains limited, with a handful of slots and insufficient funding (12). Without wider adoption of such programs, Latin America risks missing the opportunity to develop its new generation of physician-scientists equipped to address the region’s complex health challenges.

#### 3.1.1 Recommendations for improvement

1. *Establish Regional Dual-Degree Programs:* Countries across Latin America should collaborate to create structured, dual-degree programs that combine medical education with research training. Regional networks could share resources and create exchange programs to broaden opportunities for aspiring physician-scientists.
2. *Reform and Harmonization of Medical Curricula:* Incorporating research methodologies, data analysis, and critical thinking into the core curriculum of medical schools is essential. This approach would ensure that all medical students gain a foundational understanding of research, even if they do not pursue it as a career.
3. *Strengthen and Regulate Scientific Initiation Programs:* More structured programs should be developed with enhanced support for mentors, ensuring a consistent quality of research experience. Additionally, regional funding agencies could coordinate efforts to harmonize the curricula, support cross-country mentorship initiatives, and promote feedback mechanisms from mentees.

### 3.2 Post-graduation: systemic challenges

Upon graduation, early career physicians encounter a fragmented landscape where the paths of medical residency, specialization, and research rarely converge and often compete. In countries like Argentina, Brazil, Mexico and Peru, medical residency is the default route, but it rarely leads to a career that effectively combines clinical practice with research, due to systemic barriers.

Public healthcare systems across Latin America are often overburdened and underfunded, adding pressure on residents, who are often expected to focus solely on clinical responsibilities. With intense workloads and relatively low stipends, residents often work extra shifts during off-hours to manage living expenses (14, 15). For instance, medical residents in Argentina and Mexico often earn less than their counterparts in developed countries, making it financially unsustainable to prioritize research over clinical duties (16, 17).

Research fellowships that align with clinical training are scarce and often require exclusivity, prohibiting fellows from supplementing their income. This is challenging, as stipends are often very low, failing to account for fellows’ advanced training and professional experience. Although recent efforts in Peru have sought to integrate research fellowships with clinical training through partnerships with international centers, these opportunities remain limited and are concentrated in Lima limiting the reproducibility of the experience and leaving other regions underserved (18, 19). Similarly, Uruguay has introduced structured initiatives to promote early research engagement. Since 2008, its largest public medical school has run a mandatory scientific methodology course for final-year students, reaching over 600 students and 300 faculty annually and credited with launching numerous academic careers (20, 21). In Colombia, the growing focus on research has led to post-graduate programs with some research integration, but the lack of protected time for research remains a significant barrier (14).

Mentorship within this context is equally strained. With no standardized training framework, mentors are often forced to design “on-the-go” training regimens, leading to inconsistencies in the quality and depth of research experiences. A recent study in the Latin American oncology community highlights the urgent need for structured mentor-mentee programs, showing their benefits for career development and research productivity (22). This reinforces the importance of regionally adapted, institutionalized mentorship frameworks across medical specialties. However, designing these tailored programs demands significant time and effort, detracting from mentors’ own research and clinical responsibilities. Effective mentorship ideally involves structured schedules, hands-on research opportunities, and guidance on balancing dual roles. Yet mentors frequently go beyond academic support, sometimes even providing financial assistance to their mentees.

The role of preceptors in scientific training remains incipient and undervalued in Latin America, with only a small proportion of professionals possessing the expertise and access to resources needed to fully support trainee development (3). Often overwhelmed by their responsibilities, these mentors juggle their professional and research obligations while striving to offer the necessary guidance and opportunities for their trainees to succeed.

#### 3.2.1 Recommendations for improvement

1. *Develop Integrated Residency-Research Programs:* Creating structured programs that balance clinical training with dedicated research tracks, such as MSc pathways, would help reduce the gap in formal research training. Such programs should provide funding for research projects and protected time for scientific work.
2. *Align Graduate Programs with Clinical Duties:* Adjusting the schedules of master’s and doctoral programs to fit the demanding work hours of physicians could make research training more accessible.
3. *Recognize Research Achievements:* Introducing awards and incentives for physician-scientists who excel in research can help shift the cultural perception of dual careers in the medical community.
4. *Enhance Mentorship Support:* Offering training for mentors and compensating them for their time can improve the quality of mentorship, fostering a more consistent and supportive environment for trainees. Partnerships with international organizations could help fill this gap, offering formal mentorship training and exchanges. Incorporating mentorship training within grants, or as a requirement of certain fellowship programs, could provide structured support to enhance mentorship quality across the region.
5. *Establish Patient-Oriented Research Career Development Programs:* Creating mentored career development programs, such as patient-oriented research tracks funded by Latin American research agencies, would help cultivate the next generation of physician-scientists. Modeled after initiatives like the K23 awards from the NIH, these programs would provide physician-scientists with dedicated funding and structured mentorship in conducting patient-centered research.

## 4 Institutional and funding challenges

Beyond education, Latin America’s physician-scientists face significant institutional and funding barriers. While institutions like the Instituto Nacional de Salud in Peru, the Universidad de la República in Uruguay, and the Universidad Nacional in Colombia are known for their research output, many universities and research centers across the region lack the necessary infrastructure and resources to support a robust research environment (6). This disparity creates uneven opportunities, where some countries or regions can cultivate research talent while others struggle to retain even their most promising scientists (23, 24).

State-level research funding agencies in some countries provide significant support but highlight the regional disparities even within a single country.

Brazil’s state-level research funding agencies, such as the São Paulo Research Foundation (FAPESP) and the Carlos Chagas Filho Foundation for Research Support of the State of Rio de Janeiro (FAPERJ), provide crucial support but highlight the regional disparities even within a single country (25, 26). In Argentina the National Scientific and Technical Research Council (CONICET), and in Mexico, the National Council of Humanities, Science and Technology, (CONAHCYT) play similar roles but face challenges with inconsistent funding and shifting political priorities (27, 28). Moreover, postdoctoral fellowships in Latin America still come with low stipends and conditions that discourage participation in additional research projects, making it difficult for scientists to maintain a steady income while pursuing their work (6).

International models, such as those offered by the National Institutes of Health (NIH), Wellcome Trust and UK Research and Innovation, provide competitive funding, extensive research resources, and access to broad professional networks. However, the educational challenges are compounded by insufficient administrative and institutional support in Latin America, and hampers researchers’ competitiveness. Despite these challenges, the most skilled or well-positioned researchers often find success, which leads to a brain drain, as those who relocate abroad have few incentives to return, with weak positions and unstable, short-duration contracts (29).

There are several successful models that Latin America could adopt. Programs like the NIH’s T32 and T35 grants schemes in the United States provide a well-structured framework that supports the transition from training to independent research careers by offering stipends, tuition assistance, and research funding, while also requiring the applicant institutions to have good scientific infrastructure, a detailed training plan and dedicated mentors. Similarly, initiatives such as the physician-scientist Training Programs present an integrated approach to combining clinical training with research, providing a clear roadmap and educational resources for career development that includes funding opportunities with protected time for research during residency.

### 4.1 Recommendations for improvement

1. *Increase Funding and Eliminate Restrictive Work Requirements*: Raising stipends for research fellowships and removing exclusivity clauses, which prohibit fellows from holding additional paid positions during their training, would make research careers more financially viable and appealing to young scientists.
2. *Create Clear Career Development Pathways:* Establishing well-defined career tracks with mentorship programs, workshops, and milestones could provide clarity and direction for physician-scientists, helping them navigate their careers more effectively.
3. *Attract and Retain Talent:* Competitive salaries, start-up grants for laboratories, and programs to facilitate the return of scientists working abroad are crucial to reversing the trend of talent loss in the region.

## 5 Collaborative opportunities and the way forward

For Latin America, the training and retaining physician-scientists are not merely academic exercises; they are critical to the region’s ability to respond to current and future health challenges. Rapid advancements in AI, precision medicine, and host-directed therapies, alongside the ongoing need for pandemic preparedness, demand investment in a new generation of physician-scientists who can lead innovation (30–32). Addressing the systemic barriers to dual careers in medicine and research is vital to ensuring that the region remains competitive on the global stage. As highlighted in Table 1, the levels of support, research integration, and challenges faced by physician-scientists vary widely across key Latin American countries, underscoring the need for a cohesive, regional approach to support these professionals and to address existing disparities through collaborative efforts.

**TABLE 1:** Comparison of key features in medical curricula, research policies, and incentives for physician-scientists across Latin American countries.

Country	Medical curriculum duration	Integration of research in curriculum	Key research funding programs	National research support initiatives	Mentorship structures	Dual-degree programs (e.g., MD-PhD)	Challenges
Brazil	6 years	Limited integration, with scientific initiation programs (CNPq)	FAPESP, CNPq, FAPERJ	Fiocruz research initiatives	Limited, often informal	Rare but emerging (e.g., USP initiatives)	High regional disparity, brain drain
Peru	6 years	Early exposure through CONCYTEC programs	CONCYTEC, private sector partnerships	National and international research collaborations	Limited regional coverage	In development phase	Centralization in Lima, lack of consistent mentorship
Colombia	6 years	Pilot MD-PhD programs, regional variation	COLCIENCIAS, private funding	University-led research initiatives	Emerging, but variable	Limited, often pilot programs	Underfunding, lack of protected research time
Uruguay	6 years	Focused in public universities, limited in private	ANII	Research collaboration networks (LACRN)	Informal, reliant on faculty initiatives	Not yet established	Small academic community, funding constraints
Argentina	6 years	Programs through CONICET and universities	CONICET, provincial programs	Partnerships with international research bodies	Limited, variable quality	Rare, mostly independent initiatives	Political instability affecting funding
Mexico	6 years	Varies widely across institutions	CONACYT	National Researcher System (SNI)	Structured but unevenly distributed	Few, mostly in major universities	Economic challenges, high clinical workloads

The absence of regional initiatives designed to support, finance, and foster the development of physician-scientists creates isolated pockets of progress rather than a cohesive regional strategy. While Brazil has its CNPq fellowships and Peru supports research through CONCYTEC, there is little coordination or shared vision across borders. This fragmentation results in duplication of efforts, inefficient use of resources, and missed opportunities for collaborative research and training. Initiatives like the IeDEA network, which includes several Latin American countries in HIV research, exemplify how shared resources and collaborative studies can advance scientific understanding (33). Serving as inspiration, Africa’s Alliance for Accelerating Excellence in Science in Africa (AESA) provides funding, mentorship, and continent-wide networks, while Europe’s Marie Skłodowska-Curie Actions (MSCA) facilitate cross-country researcher mobility and training, fostering collaboration across European nations.

Latin America could benefit greatly from creating a regional consortium dedicated to training physician-scientists. A Latin American consortium could be coordinated through regional scientific bodies and supported by a coalition of national research agencies. Practical components might include harmonized research training curricula, regional fellowship calls with co-funded stipends, and structured mentorship exchanges between institutions in different countries. Leveraging virtual platforms could further reduce costs and increase accessibility, while existing international funding mechanisms, such as those offered by the NIH’s Fogarty International Center or the Wellcome Trust, could be engaged to support pilot phases. More importantly, this approach could enhance the collective capacity of Latin America’s medical and scientific community, enabling it to respond more effectively to global health challenges.

The region’s diverse healthcare challenges, from tropical diseases in the Amazon basin to the high prevalence of metabolic disorders in urban centers, also offer an ideal context for reciprocal innovation. This approach, already promoted by organization like the Fogarty International Center, supports a two-way exchange of solutions, where insights gained in resource-limited settings inform practices globally, and advances from high-resource environments are adapted to meet local needs, building equity and fairly developing local research communities (33–35).

Furthermore, the rise of private medical institutions in Latin America offers a new frontier for innovation. Historically, scientific production in the region has been concentrated in public universities, but private entities are increasingly investing in research. This shift presents a chance to redefine medical training and introduce more structured programs that align with global standards (36, 37). Notably, initiatives such as the Latin American Alliance of Academic Health Centers and consortia linked to international collaborations, such as the Fogarty International Center programs and the IeDEA network, illustrate how public-private and cross-border efforts can support research capacity-building and mentorship across the region. These collaborations have enabled data-sharing, multicenter studies, and the establishment of training hubs that benefit both public and private institutions. However, this potential can only be realized through a long-term commitment to excellence and collaboration between private and public sectors to prevent exacerbating existing inequalities.

Considering both the feasibility and prioritization of proposed reforms, in the short term, the greatest impact may come from removing barriers and offering clear incentives. Eliminating exclusivity clauses in fellowships, for instance, would allow trainees to supplement their income and reduce financial strain, making research careers more accessible. Likewise, compensating mentors for their time and formally valuing their contribution would elevate the quality and consistency of mentorship while expanding the pool of willing mentors. These reforms require limited structural changes and are more readily actionable within current institutional and funding frameworks. By contrast, long-term initiatives, such as integrating research into clinical residency programs, launching dual-degree pathways, or establishing a regional consortium, are transformative but require sustained policy coordination and investment. A pragmatic approach involves addressing these priorities in parallel: implementing low-barrier, high-yield reforms immediately, while building momentum for structural innovations through regional and international collaboration.

Embracing these changes will allow Latin America’s physician-scientists to lead the transformation of healthcare in their countries, driving innovation that can serve as a model for other regions facing similar challenges. A united, regional effort would not only improve training and research opportunities but also ensure that Latin America contributes to global advancements in medicine and public health.
